# CD146^+^ mesenchymal stem cells display greater therapeutic potential than CD146^–^ cells for treating collagen-induced arthritis in mice

**DOI:** 10.1186/s13287-016-0285-4

**Published:** 2016-02-03

**Authors:** Cheng-Chi Wu, Fei-Lan Liu, Huey-Kang Sytwu, Chang-Youh Tsai, Deh-Ming Chang

**Affiliations:** Graduate Institute of Life Sciences, National Defense Medical Center, No.161, Sec. 6, Minquan E. Rd., Neihu Dist., Taipei, 114 Taiwan Republic of China; Taipei Veterans General Hospital, No.201, Sec. 2, Shipai Rd., Beitou District., Taipei, 112 Taiwan Republic of China

**Keywords:** CD146, Mesenchymal stem cells, Collagen-induced arthritis

## Abstract

**Background:**

The characteristics and therapeutic potential of subtypes of mesenchymal stem cells (MSCs) are largely unknown. In this study, CD146^+^ and CD146^–^ MSCs were separated from human umbilical cords, and their effects on regulatory T cells (Tregs), Th17 cells, chondrogenesis, and osteogenesis were investigated.

**Methods:**

Flow cytometry was used to quantify IL-6 and TGF-β1 expressed on CD146^+^ and CD146^–^ MSCs. The therapeutic potential of both subpopulations was determined by measuring the clinical score and joint histology after intra-articular (IA) transfer of the cells into mice with collagen-induced arthritis (CIA).

**Results:**

Compared with CD146^–^ MSCs, CD146^+^ MSCs expressed less IL-6 and had a significantly greater effect on chondrogenesis. After T lymphocyte activation, Th17 cells were activated when exposed to CD146^–^ cells but not when exposed to CD146^+^ cells both *in vitro and in vivo*. IA injection of CD146^+^ MSCs attenuated the progression of CIA. Immunohistochemistry showed that only HLA-A^+^ CD146^+^ cells were detected in the cartilage of CIA mice. These cells may help preserve proteoglycan expression.

**Conclusions:**

This study suggests that CD146^+^ cells have greater potency than CD146^–^ cells for cartilage protection and can suppress Th17 cell activation. These data suggest a potential therapeutic application for CD146^+^ cells in treating inflammatory arthritis.

## Background

Rheumatoid arthritis (RA) is a chronic destructive polyarthritis. Disease progression induces the production of lesions in joints, which can cause severe damage that leads to cartilage destruction and bone erosion, and eventually to the loss of joint function [1, 2]. Synovial fluid and the serum of RA patients contain high levels of proinflammatory cytokines such as interleukin (IL)-1β, IL-6, and tumor necrosis factor alpha (TNFα) [3, 4]. TNFα in joints is produced mainly by macrophages and can induce cartilage degradation and antigen-presenting cells, which can trigger IL-17-producing effector T helper cells (Th17) activation in RA [5, 6]. Th17 cells play an important role in the progression of RA, and Th17 cells and IL-17 are both highly correlated with disease activity in RA. IL-17 can induce the release of proinflammatory cytokines and matrix metalloproteinases by synovial fibroblasts [7, 8]. Recent studies have shown that blocking IL-17 release can control the disease progression and joint destruction in an animal model of inflammatory arthritis [9, 10].

Mesenchymal stem cells (MSCs) may affect tissue repair in RA because of their multipotent differentiation capacity and their ability to modulate immune responses [11, 12]. Based on the International Society for Cellular Therapy criteria, MSCs can differentiate into osteoblasts, adipocytes, chondroblasts, and chondrocytes in vitro [13]. Recent studies have shown that MSCs suppress immune cell functions directly by releasing transforming growth factor beta (TGF-β) and IL-10 or indirectly by inducing regulatory T (Treg) cells in vitro and in vivo [11, 14–16].

CD146 (also known as melanoma cell adhesion molecule) is an antigen that is expressed on almost all kinds of epithelial cells, activated T cells, and dendritic cells [17–19]. The functions of CD146 remain elusive. It is presumed that CD146 is involved in regulating cell mobility, and might be involved in adhesive interactions binding to CD146 receptors and ERM (ezrin, radixin and moesin) proteins [20, 21]. CD146 has been identified as an early mesenchymal marker present in MSCs obtained from human bone marrow, dental pulp, adipose tissues, and the umbilical cord [22–25]. CD146^+^ stem cells have greater multilineage potency and in vitro immunomodulatory effects compared with CD146^–^ cells, and this subpopulation may be a candidate marker for MSC isolation [26–29].

Our previous study showed that human stem cells derived from umbilical cords may not be therapeutic for RA because human umbilical cord-derived mesenchymal stem cells (HUMSCs) induce IL-17 and Th17 cells in TNFα-dominant situations [30]. To understand the potentially different outcomes of MSCs used in stem cell therapy, we hypothesized that these differences may be attributed to the existence of distinct subpopulations in the isolated stem cells. During RA progression, cartilage degradation and bone erosion cause irreversible damage. Thus, intra-articular (IA) injection of MSCs may be an effective technique for inducing the local repair of joint destruction [12, 31]. The main point of this study was using the IA injection method to collect possible direct evidence for tissue repair. We assume IA injection might be a possible therapeutic option in the future. In this study, we investigated the disease progress and joint histological change in arthritis mice after IA injection of CD146^+^ cells and CD146^–^ cells. To our knowledge, this is the first study to show the role of CD146 antigen in the immunomodulatory effects of MSCs. Our results show that CD146 antigen is a marker of progenitor cells and a suppressor of Th17 cells, and plays a role in promoting chondrogenesis. Our data suggest that these cells have therapeutic potential for treating RA.

## Methods

### Patients’ samples

The protocol for the sampling of human umbilical cords was approved by the Institutional Review Board of the Tri-Service General Hospital, National Defense Medical Center, Taiwan, Republic of China (Protocol No. 097-05-0005). The participants signed an informed consent form before participating.

The protocols for the collection and expansion of HUMSCs were as described in our previous study [30]. Briefly, umbilical cords were obtained from consenting patients who delivered a full-term infant by cesarean section. After the arteries and veins were removed, the remaining tissue was transferred to fresh growth medium. The cells were left undisturbed for 5–7 days in a 37 °C humidified incubator with 5 % carbon dioxide (CO_2_) to allow migration of cells from the explants, after which the medium was replaced every 2 days. When cells reached 80–90 % confluence they were harvested using 0.05 % trypsin–0.53 mM EDTA solution and frozen at –80 °C until use in the experiments.

The protocol for sampling human synovial fluid was approved by the Institutional Review Board of the Tri-Service General Hospital, National Defense Medical Center, Taiwan, Republic of China (Protocol No. 2-101-05-108). The participants signed an informed consent form before participation. Synovial fluid was obtained from five patients with RA. All patients met the American College of Rheumatology 1987 revised criteria for RA [32]. To avoid possible interindividual differences between patients, the synovial fluid samples from the five patients were pooled together and centrifuged at 1600 xg for 15 minutes to remove cartilage. The combined synovial fluid was stored at –80 °C until use.

### Isolation and identification of CD146 stem cells

Before the CD146 sorting, the expression of stem cell markers and the differentiation potential of the HUMSCs were determined to exclude the effects of other major stem cell markers [30]. CD146^+^ cells were collected and isolated from human umbilical cord-derived stem cells using a BD IMag Magnetic Cell Separation System (BD Biosciences, San Diego, CA, USA). Biotin-conjugated CD146 antibody (P1H12; Abcam, Cambridge, MA, USA) was used to sort CD146^+^ cells. Twelve to 25 % of the total HUMSCs were CD146^+^. The protocol for cell isolation followed the instructions for the BD IMag Cell Separation System. Briefly, the cells were cultured with anti-CD146 antibody for 30 minutes at 37 °C, added to BD IMag Streptavidin Particles Plus-DM and incubated for 30 minutes at 37 °C, and sorted using the BD IMagnet. The sorted cells were cultured in growth medium to a sufficient quantities for further research. Duplications of isolated MSCs from passages 3–7 were used for the experiments. The doubling time of CD146 cell subpopulations was calculated using the Doubling Time algorithm(http://www.doubling-time.com/compute.php).

### Enzyme-linked immunosorbent assay to measure 5-bromo-2′-deoxyuridine incorporation

For the 5-bromo-2′-deoxyuridine (BrdU) enzyme-linked immunosorbent assay (ELISA; Roche, Indianapolis, IN, USA), the protocol followed the manufacturer’s instructions. Briefly, 1 × 10^3^ or 5 × 10^3^ cells were loaded into a 96-well plate with Dulbecco’s modified Eagle’s medium (DMEM 11885, Gibco, Gaithersberg, MD, USA) containing 10 % fetal bovine serum (FBS, Invitrogen, Carlsbad, CA, USA), the plate was incubated overnight, BrdU-labeling dye was added, and the plate was incubated for 0, 2, or 4 hours. The cells were incubated with BrdU–peroxidase-linked monoclonal antibody for 90 minutes, and tetramethylbenzidine staining was detected. Cell proliferation was measured at 450 nm in an ELISA reader.

### Chondrogenesis and osteogenesis of HUCSCs

To analyze the effects of inflammation on chondrogenic and osteogenic capacities, the CD146^+^ and CD146^–^ cells were cultured in chondrogenic and osteogenic medium. Then saline, 10 ng/ml TNFα, or 10 % synovial fluid from patients with RA (RASF) was added to the two differential medium.

For the chondrogenesis experiment, 5 × 10^5^ MSCs were trypsinized and cultured in 1 ml complete DMEM in 15 ml polypropylene conical tubes. The FBS-containing medium was replaced with 1.5 ml basal medium containing low-glucose DMEM, 100 U/ml penicillin, 100 μg/ml streptomycin, 1 mM sodium pyruvate, 1 % insulin- transferrin- selenium (Sigma Aldrich, St. Louis, MO, USA), 100 nM dexamethasone (Sigma Aldrich), 50 μg/ml ascorbic acid (Sigma Aldrich), 5.35 mg/ml linoleic acid (Sigma Aldrich), and 1.25 mg/ml bovine serum albumin (Sigma Aldrich) with or without 10 ng/ml TGF-β3 (PeproTech EC, London, UK). For quantification, the Safranin O-stained pellets were measured on a slide using US National Institutes of Health Image software at 100× magnification (ImageJ, http://imagej.nih.gov/ij/).

For the osteogenesis experiment, 6 × 10^4^ MSCs were added to a 24-well culture plate containing osteogenesis medium. The medium was replaced every 3 days. The osteogenesis medium contained high-glucose DMEM, 100 U/ml penicillin, 100 μg/ml streptomycin, 10 mM β-glycerophosphate (Sigma Aldrich), 100 nM dexamethasone (Sigma Aldrich), and 50 μg/ml ascorbic acid (Sigma Aldrich). After 2 weeks, the differentiated cells were stained with Alizarin Red S (Sigma Aldrich) to detect osteogenesis. For quantification, the Alizarin Red S-stained slides were measured on a slide using US National Institutes of Health Image software at 100× magnification [33].

### Animals

All animal experiments were approved by the Animal Care and Use Committee of the National Defense Medical Center, Taiwan, Republic of China (IACUC-08-032). DBA/1J mice aged 7 weeks were purchased from the Jackson Laboratory (Bar Harbor, ME, USA) and housed under specific pathogen-free conditions in the Laboratory Animal Center of the National Defense Medical Center. All animals were housed individually and monitored daily. The mice were euthanized by inhalation of CO_2_ 14 days after arthritis onset, and the joints, spleens, and lymph nodes were harvested for further studies.

### Lymph node isolation and MSC–T cell coculture

Lymph nodes were isolated from healthy male DBA/1J mice. The mice were euthanized by inhalation of CO_2_, and the lymph nodes and spleen were harvested and crushed in phosphate-buffered saline (PBS). To isolate the cells, the suspension in 4 ml PBS was slowly placed on 8 ml Ficoll–Hypaque, the mixture was centrifuged at 500 × *g* for 30 minutes, and the cells were collected and suspended in RPMI 1640.

To determine the effects of MSCs on T cells, 10^5^ MSCs were treated with or without 10 ng/ml TNFα for 24 hours, and then stimulated with 1 × 10^6^ splenocytes with phytohemagglutinin-L (Sigma Aldrich) and 1 μg/ml anti-CD146 antibody in RPMI 1640 containing 10 % FBS. After 2 days, the suspended cells were harvested and Th17 and Treg cells were identified by flow cytometry. The supernatants from MSC–T cell cocultures were harvested and detected the cytokine levels for an ELISA assay. The antibodies used were fluorescein isothiocyanate (FITC)-conjugated rat anti-mouse CD4 (eBioscience, San Diego, CA, USA), phycoerythrin (PE)-conjugated rat anti-mouse IL-17A (eBioscience), and PE-conjugated rat anti-mouse Foxp3 (eBioscience). Analyses were performed on a FACSort cytometer using CellQuest software (BD Bioscience).

### Measurement of immunomodulatory cytokines

The intracellular cytokines were detected by flow cytometry. For intracellular staining, cells were permeabilized using a BD Fixation/Permeabilization kit (BD Bioscience). The antibodies used were FITC-conjugated anti-human IL-6 (eBioscience), PE-conjugated anti-human TGF-β1 (BioLegend, San Diego, CA, USA), and PE-conjugated anti-human IL-10 (eBioscience). Analyses were performed on a FACSort cytometer using CellQuest software (BD Bioscience). Immunotyping was detected according to our previous study [30].

To measure the secretions of human IL-6 and TGF-β1 on TNFα- treating MSCs, MSCs were treated with or without 10 ng/ml TNFα for 3 days. The concentration of these cytokines was measured in the supernatants using Platinum ELISA kits (eBioscience) and murine IL-10 and IL-17 ELISA kits (R&D Systems, Minneapolis, MN, USA). All of the samples from cocultured supernatants or serum were quantified according to the manufacturer’s instructions.

### Induction of the collagen-induced arthritis model

Five independent immunized mice were analyzed in each group. To determine the effects of CD146^+^ and CD146^–^ cells in arthritic mice, each mouse’s hind limb was given an IA injection of 10^6^ cells after the appearance of joint swelling in the same mice. The collagen-induced arthritis (CIA) mice were given an IA injection of saline as control. To avoid individual variation, the same offspring were injected intra-articularly at the same arthritis scores (arthritis score = 3) in all groups.

We used the same protocol as in our previous study [30]. Briefly, 8-week-old male DBA/1 mice were immunized by subcutaneous injection into the tail with 100 μg bovine type II collagen emulsified in Freund’s complete adjuvant (Chondrex, Redmond, WA, USA). After 21 days, a booster intradermal injection of the tail was given with 100 μg bovine type II collagen emulsified in Freund’s incomplete adjuvant (Chondrex). Paw swelling began 21–28 days after immunization. Upon appearance of the signs of arthritis, defined as severe swelling, each mouse was given an IA injection of 10^6^ cells or saline control. Fourteen days after IA injection, the mice were euthanized by inhalation of CO_2_, and the joint tissues were fixed for further studies. The arthritis signs were scored as clinical signs of inflammation: 0 = normal, 1 = slight swelling, 2 = moderate swelling, 3 = severe swelling and reversible joint immobility, and 4 = severe swelling and irreversible joint immobility.

### Histological staining

Immunohistochemical staining for human leukocyte antigen (HLA-A) and IL-17 was performed using heat-induced antigen retrieval with Dako REAL™ Target Retrieval Solution (Dako, Carpinteria, CA, USA). Paraffin sections were treated with goat blocking serum for 20 minutes and then incubated with primary antibodies. Primary antibodies against human HLA-A (A-18) and IL-17 (H-132) were purchased from Santa Cruz Biotechnology (Dallas, TX, USA) and antibodies against human CD146 (P1H12) were purchased from Abcam. Sections were incubated with primary antibodies at 4 °C overnight and then incubated for 1 hour with bovine anti-goat FITC–IgG or bovine anti-rabbit rhodamine–IgG (Santa Cruz Biotechnology). Fluorescence was detected on a Leica fluorescence microscope LeicaDMI6000B (Wetzlar, Germany).

To identify cartilage degradation, tissue sections were stained with 0.05 % (w/v) Fast Green (Sigma) for 5 minutes, washed quickly in 0.1 % acetic acid, and then stained with Safranin O (Sigma) for 5 minutes. The cartilage degradation score from 0 to 3 was defined as either no loss of proteoglycans or complete loss of staining for proteoglycans.

### Statistical analysis

Each experimental group had five independent samples. Mean ± standard error of the mean (SEM) values were calculated, and the significance of differences was calculated using Tukey’s test to compare pairs in an analysis of variance. *P* <0.05 was considered significant.

## Results

### Morphology and proliferation of CD146^+^ and CD146^–^ MSCs

**Fig. 1:**
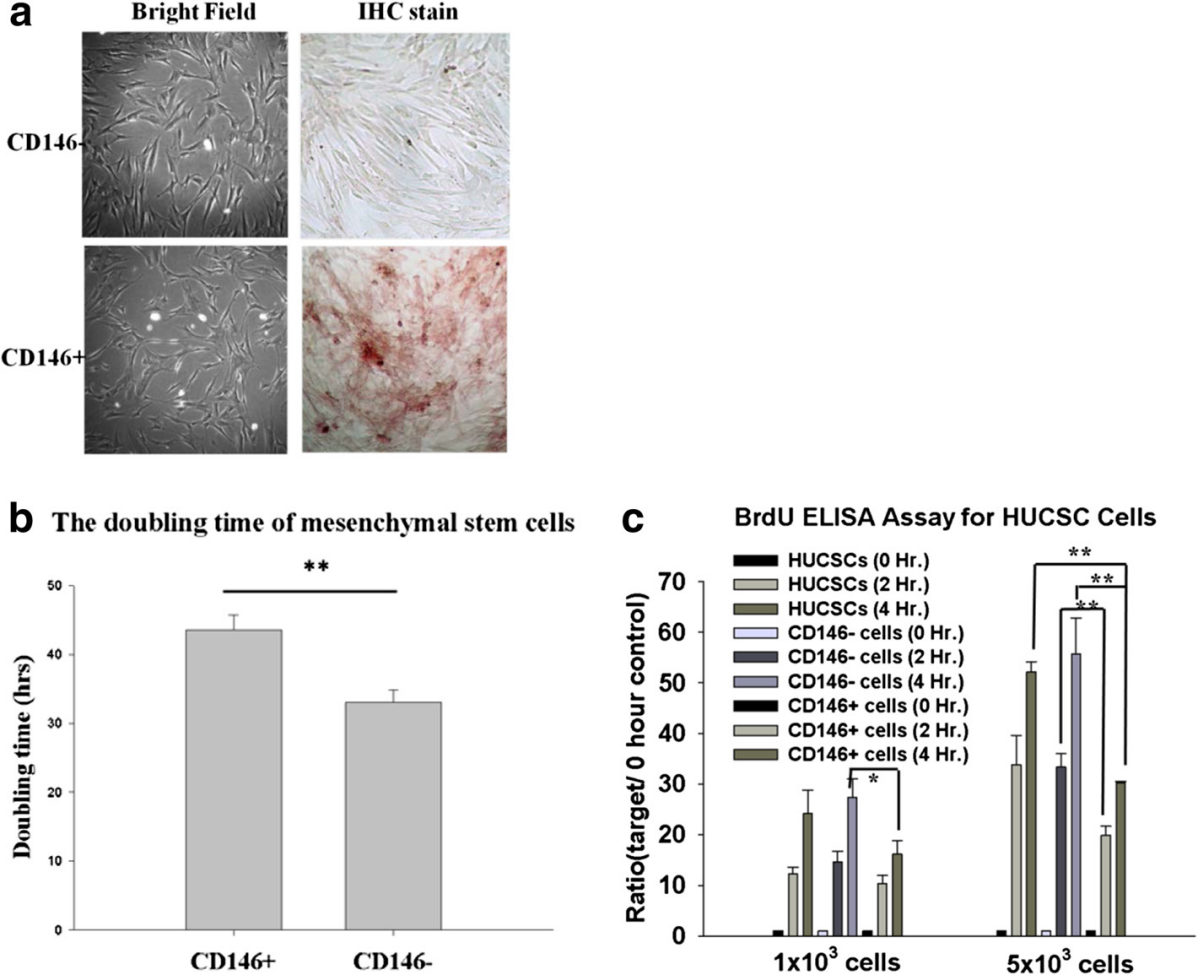
Phenotypes of expanded CD146^+^ and CD146^–^ MSCs, and their proliferative properties. The phenotypes and anti-CD146 immunohistochemical staining of CD146^+^ and CD146^–^ cells (magnification, 100×). **a**–**c** The proliferation rates of CD146^+^ and CD146^–^ cells were detected by their doubling time and analyzed by BrdU ELISA. Data are expressed as mean ± SEM from five independent experiments. *P <0.05, **P <0.01. *BrdU* 5-bromo-2′-deoxyuridine, *ELISA* enzyme-linked immunosorbent assay, *Hr* hours, *IHC* immunohistochemistry

The isolated CD146^+^ and CD146^–^ MSCs showed similar morphology, but only CD146^+^ cells exhibited CD146 on the cell surface (Fig. 1a). The fast growth in CD146^–^ MSCs was confirmed by the shorter doubling time and higher proliferation rate (Fig. 1b, c). Figure 1b shows that the doubling time was significantly longer in CD146^+^ cells than in CD146^–^ cells. A BrdU ELISA showed significant differences between CD146^+^ cells and CD146^–^ cells after 2 hours and 4 hours of culture (Fig. 1c; 1 × 10^3^ cells, *P* <0.05; 5 × 10^3^ cells, *P* ≤0.001). Moreover, there was a significant difference in growth rate between HUCSCs (human umbilical derived mesenchymal stem cells) and CD146^+^ cells after 4 hours of culture (Fig. 1c; 5 × 10^3^ cells, *P* ≤0.001).

### Chondrogenesis and osteogenesis of CD146^+^ and CD146^–^ MSCs

**Fig. 2:**
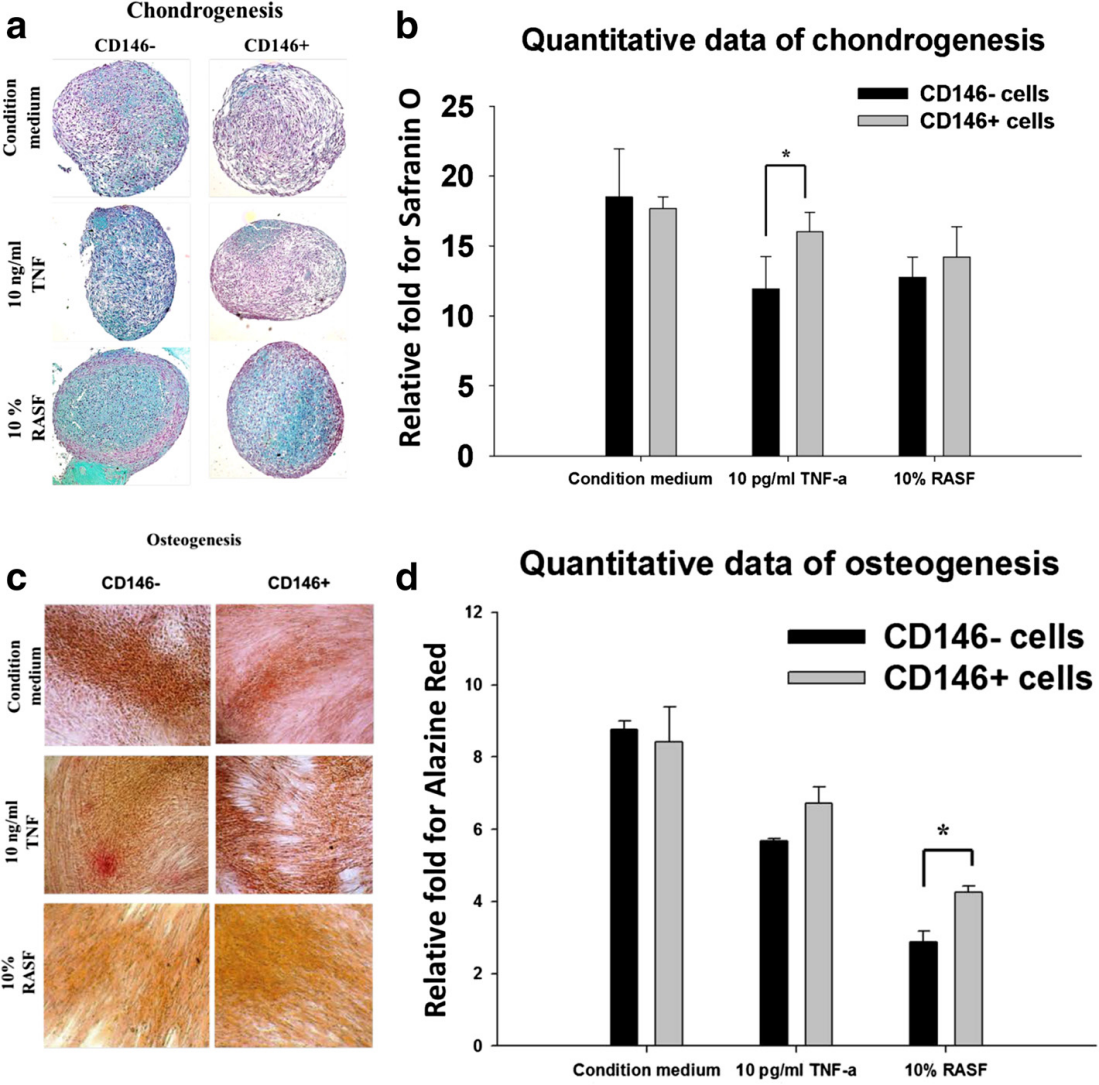
In vitro differentiation potential of CD146^+^ and CD146^–^ MSCs. Isolated MSCs were cultured in chondrogenesis or osteogenesis condition medium, and then treated with 10 ng/ml TNFα or 10 % synovial fluid from RA patients. The cells were stained with Safranin O **a**, **b** and Alizarin Red S **c**, **d** to detect the differentiation potential for chondrogenesis and osteogenesis, respectively. Quantitative data for chondrogenesis and osteogenesis are expressed as mean ± SEM compared with the nondifferentiation-negative control. *P <0.05. *RASF* synovial fluid from rheumatoid arthritis patients, *TNFα* tumor necrosis factor alpha

The chondrogenic and osteogenic potential did not differ significantly between CD146^+^ and CD146^–^ MSCs. However, in isolated MSCs cultured in conditioned medium containing 10 ng/ml TNFα or 10 % RASF, CD146^–^ cells exhibited significantly less chondrogenic and osteogenic differentiation compared with CD146^+^ cells (Fig. 2; chondrogenesis, 10 ng/ml TNFα, *P* <0.05; osteogenesis, 10 % RASF, *P* <0.05).

### Expression of IL-6 and TGF-β in CD146^+^ and CD146^–^ MSCs

**Fig. 3:**
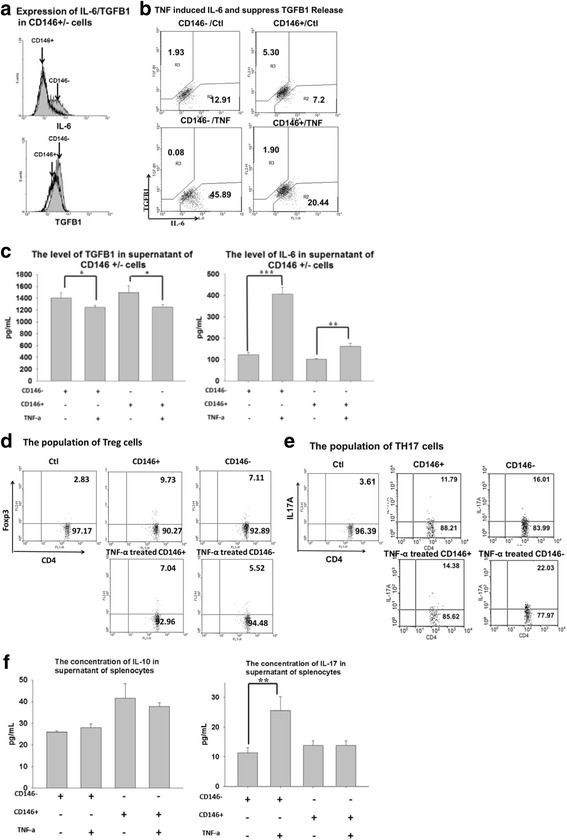
Immunomodulation of CD146^+^ and CD146^–^ cells in vitro. IL-6 and TGF-β1 expression on CD146^+^ and CD146^–^ cells was analyzed by flow cytometry and ELISA. MSCs were treated with or without 10 ng/ml TNFα for 3 days, and the concentrations of IL-6 and TGF-β1 were measured intracellularly and in the supernatants **a**–**c**. CD4^+^ Foxp3^+^ cells and CD4^+^ IL-17A^+^ cells were cocultured with TNFα-pretreated CD146^+^ cells or CD146^–^ cells for 2 days. The T cells were analyzed by flow cytometry (**d** Treg cells; **e** Th17 cells) and ELISA (**f** *left* IL-10, *right* IL-17). Data are expressed as mean ± SEM from five independent experiments. *P <0.05, **P <0.01, ***P ≤0.001. *Ctrl* control, *IL* interleukin, *TGF-β* transforming growth factor beta, *TH17* T-helper type 17, *TNFα* tumor necrosis factor alpha, *Treg* regulatory T

To examine the effects of TNFα on isolated MSCs, we used flow cytometry and an ELISA to analyze the ability of MSCs to secrete TGF-β1 and IL-6 after treatment with 10 ng/ml TNFα (Fig. 3a–c). CD146^–^ cells expressed higher levels of IL-6 than CD146^+^ cells (Fig. 3a). Treatment with TNFα inhibited the expression of TGF-β1 in both CD146^+^ and CD146^–^ cells. The number of TGF-β1-expressing cells decreased after TNFα treatment in both CD146^+^ and CD146^–^ cells. By contrast, TNFα treatment caused a significantly greater increase in the number of IL-6-expressing cells in the CD146^–^ subpopulation compared with the CD146^+^ subpopulation (Fig. 3b; *P* <0.05). The ELISA showed that TNFα inhibited the expression of TGF-β1 in both subpopulations (Fig. 3c; CD146^–^ cells, *P* <0.05; CD146^+^ cells, *P* <0.05), but promoted greater expression of IL-6 in CD146^–^ cells than in CD146^+^ cells (Fig. 3c; CD146^–^ cells, *P* ≤0.001; CD146^+^ cells, *P* <0.01).

### Effects of CD146^+^ and CD146^–^ MSCs on Treg and Th17 cells

To clarify the role of MSC subpopulations in mediating T-cell function, we analyzed the changes in Treg (CD4^+^ Foxp3^+^) cells and Th17 (CD4^+^ IL-17A^+^) cells after coculture with MSCs. Intracellular cytokines were detected in MSCs using flow cytometry, and the concentrations of IL-10 and IL-17 were measured by ELISA (Fig. 3d–f). Coculture with CD146^+^ or CD146^–^ cells had similar effects on IL-10 production by Treg cells (Fig. 3d, f left). By contrast, compared with coculture with CD146^+^ cells, coculture with CD146^–^ cells caused greater Th17 cell activation in vitro (*P* <0.05). Figure 3e, f (right) show that, compared with CD146^+^ cells, coculture with CD146^–^ cells caused significantly greater activation of the Th17 cell population (*P* <0.05) and IL-17 secretion (*P* <0.01). TNFα caused a significant increase in the Th17 cell population and IL-17A secretion in Th17 cells cocultured with CD146^–^ cells, but this effect was not seen in cocultures with CD146^+^ cells (*P* <0.01).

### Immunomodulation of CD146^+^ and CD146^–^ MSCs

**Fig. 4:**
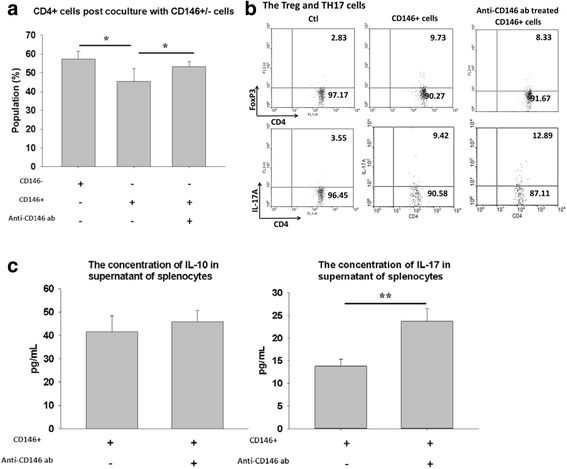
Changes in the T-cell population after coculture with CD146^+^ cells incubated with anti-CD146 in vitro. The splenocytes were cocultured with anti-CD146 antibody-treated CD146^+^ cells or CD146^–^ cells for 3 days, and the CD4^+^ cell population was measured by flow cytometry **a**. Changes in the Treg and Th17 cell populations of CD4^+^ cells were analyzed further by flow cytometry **b** and ELISA **c**. Data are expressed as mean ± SEM from five independent experiments. *P <0.05, **P <0.01 versus CD146^+^ cells alone. *ab* antibody, *Ctl* CD4^+^ T cells, *IL* interleukin, *TH17* T-helper type 17, *Treg* regulatory T

To clarify the role of CD146 antigen in mediating T-cell function, we analyzed the changes in the CD4^+^ population after coculture with MSCs for 3 days. The CD4^+^ cell population decreased significantly after splenocytes were cocultured with CD146^+^ cells but not with CD146^–^ cells or with anti-CD146 antibody alone (Fig. 4a; *P* <0.05). To investigate the effects of CD146 antigen in lymphocyte–MSC cocultures, we measured the changes in the Treg and Th17 cell populations after coculture with CD146^+^ cells that had been treated with anti-CD146 antibody. Figure 4b shows that the Th17 population increased after coculture of lymphocytes with MSCs that had been exposed to anti-CD146 antibody, but that the Treg population was not affected by antigen blocking. The ELISA also showed a significant increase in IL-17 concentration in the culture supernatants (Fig. 4c; *P* <0.01).

### Therapeutic potential of CD146^+^ and CD146^–^ MSCs in a mouse model of CIA

**Fig. 5:**
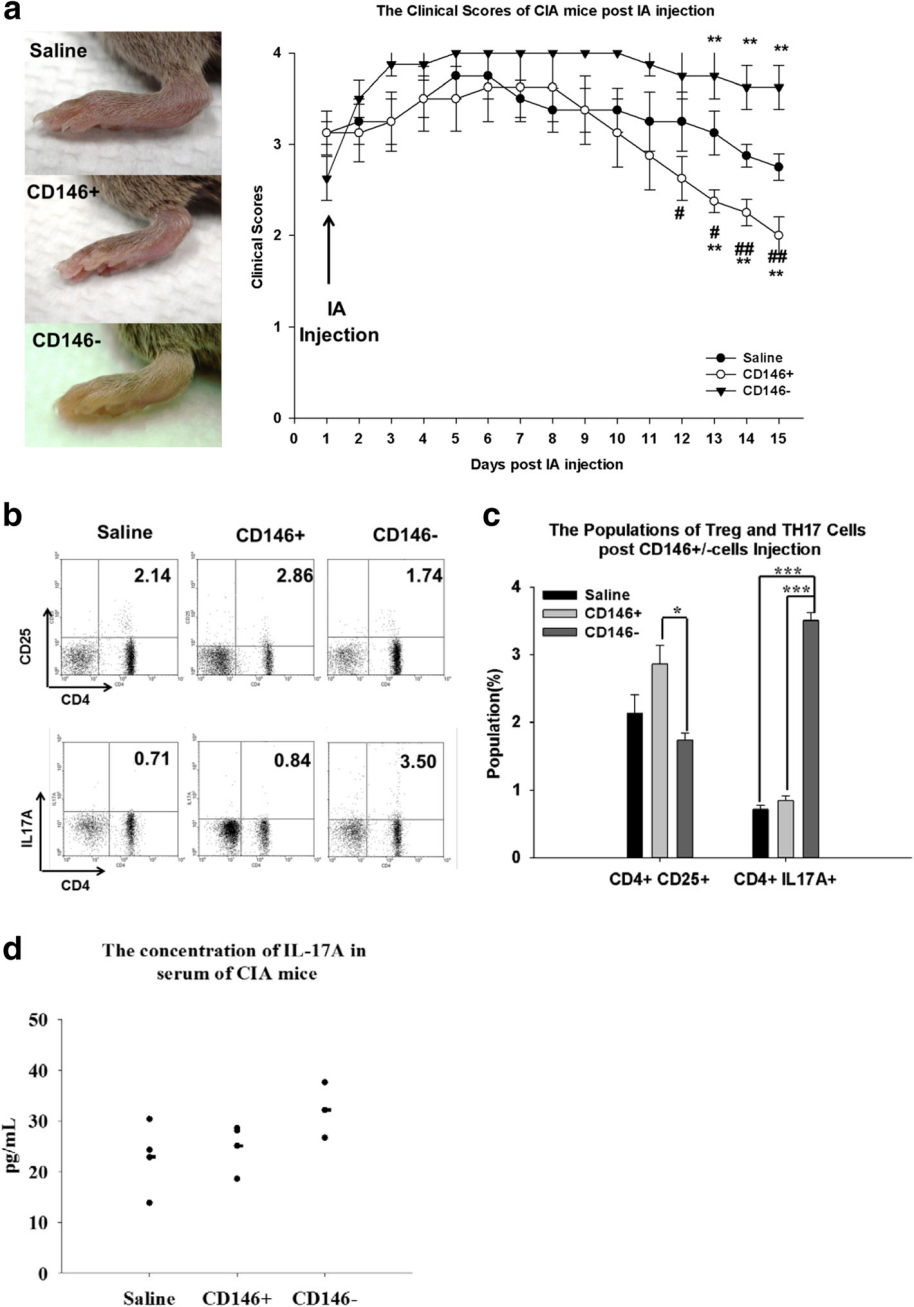
Disease progression after IA injection of CD146^+^ and CD146^–^ cells. The arthritis sign was determined as severe swelling (arthritis score = 3). IA injection of 10^6^ cells or saline control was administered to the joints of the hind paw of CIA mice. The arthritis sign and the clinical scores after IA injection of stem cells **a**. Populations of Treg and Th17 cells in CIA mice 14 days after IA injection **b**. Quantitative data for Treg and Th17 cells **c**. Serum IL-17A concentration 14 days after IA injection **d**. Data are expressed as mean ± SEM for five mice. *P <0.05 for the MSC-injected group compared with the saline control, #*P* <0.05 for the CD146^+^ cell-injected group compared with the CD146^–^ cell-injected group, **P <0.01, ***P ≤0.001, ^##^ *P* <0.01. *CIA* collagen-induced arthritis, *IA* intra-articular, *IL* interleukin, *TH17* T-helper type 17, *Treg* regulatory T

To examine the role of MSCs in joint inflammation and protection of cartilage in vivo, CIA mice with advanced arthritis in their paws were given an IA injection of isolated MSCs (clinical score = 3). Figure 5a shows that the arthritis score was significantly higher from 11 days after IA injection of CD146^–^ cells compared with IA injection of CD146^+^ cells (*P* <0.05). By contrast, the arthritis scores decreased significantly from 13 days after injection of CD146^+^ cells compared with the saline control (Fig. 5a; *P* <0.01). IA injection of CD146^–^ cells significantly worsened the disease progression from day 13 (Fig. 5a; *P* <0.01). These results suggest that CD146 subpopulations have different effects on arthritis disease progression in vivo.

To evaluate the possible mechanisms responsible for the apparent beneficial effects of CD146^+^ cells, we analyzed the influence of CD146 cells on T cells after IA injection. Contrary to our expectation, the number of Treg cells did not increase after IA injection of either CD146^+^ or CD146^–^ cells compared with the saline control. However, the population of Treg cells differed significantly between mice injected with CD146^+^ cells and those injected with CD146^–^ cells. A highly significant increase in the Th17 cell population was detected in the joints of CIA mice injected with CD146^–^ cells (Fig. 5b, c; *P* ≤0.001). To clarify the effects in Treg and Th17 cells, we also measured the concentrations of IL-10 and IL-17A in serum from CIA mice. The level of IL-17A increased after CD146^–^ cell injection in CIA mice (Fig. 5d).

### Histological staining of joints from CIA mice after IA injection of isolated MSCs

**Fig. 6:**
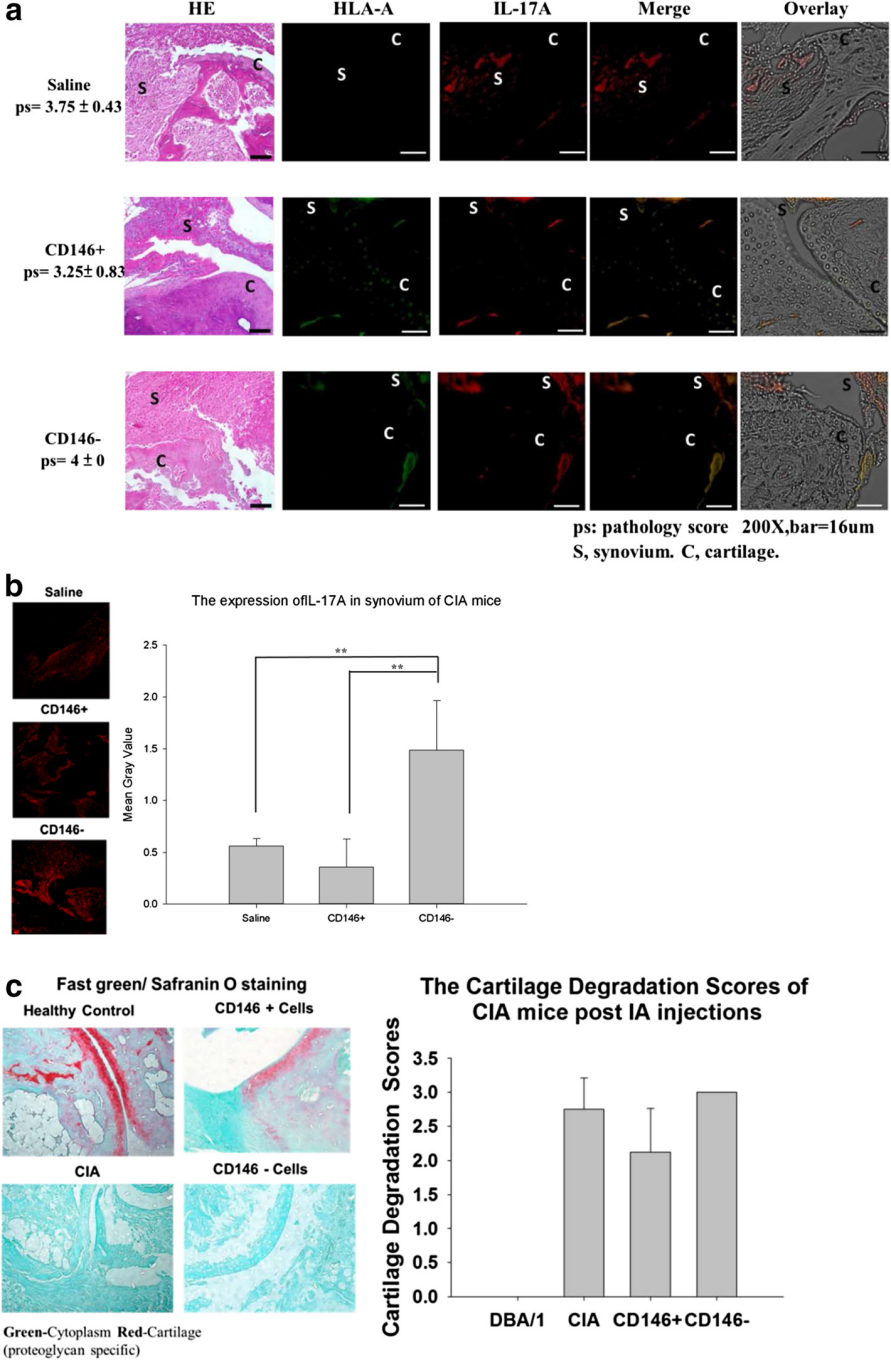
Histological and immunofluorescent (IFC) staining of HLA-A (*green*) and IL-17A (*red*) in the joints of CIA mice after IA injection of MSCs **a**. IFC staining of IL-17A (*red*) in the joints of CIA mice after IA injection of MSCs and quantification of IL-17A expression **b**. Proteoglycan expression was identified by Fast green and Safranin O staining of the joints of CIA mice after IA injection of MSC cells **c**. Data are expressed as mean ± SEM from five independent mice. *P <0.05, **P <0.01. *CIA* collagen-induced arthritis, *HE* hematoxylin and eosin stain, *HLA* human leukocyte antigen, *IA* intra-articular, *IL* interleukin

As shown in Fig. 6a, histological staining showed severe cartilage damage and bone erosion in CIA mice after IA injection of CD146^–^ cells. The pathology scores also showed more severe bone erosion in the CD146^–^ cell-injected group than in the saline or CD146^+^ cell-injected group. To track the intercellular interactions and the relationship between injected stem cells and IL-17 expression in the joints, we used immunofluorescent staining to analyze the expression of HLA-A and IL-17. CD146^+^ cells were detected in the cartilage but were seen rarely in the synovium. There was no significant difference in the serum concentrations of IL-17 between the groups of mice (Fig. 5d). As expected, IL-17 expression was higher in the synovium after injection of CD146^–^ cells compared with injection of saline or CD146^+^ cells (Fig. 6b; *P* <0.01).

To determine whether isolated MSCs can protect cartilage in vivo, the tissues were stained with Fast Green and Safranin O to detect the expression of proteoglycans in cartilage. Figure 6c shows that IA injection of CD146^+^ cells increased proteoglycan expression in cartilage, suggesting protection of cartilage, whereas injection of CD146^–^ cells did not show a similar effect.

## Discussion

Our study shows that CD146^+^ cells tended to suppress arthritis progression, as shown by their effects on chondrogenesis, IL-6 and IL-17 cytokine expression, and joint histology. CD146^–^ cells seemed to accelerate disease progression in CIA mice by inducing the proliferation of Th17 cells in vivo.

MSCs can be derived from many tissues, including bone marrow, dental pulp, adipose tissue, and umbilical cord matrix [34–37]. However, these cells show different properties, multilineage potential, and immune-mediating abilities related to their different cell niches [38, 39]. Several cell surface markers are used to identify MSC subtypes. For example, MSCs expressing CD56 and CD271 have greater capacity for differentiation than CD56^–^ or CD271^–^ MSCs even though these cells also express CD73, CD90, and CD105 [40]. Recent reports showed that the expression of CD146 by MSCs had no significant effects on MSC characters [41, 42]. We checked the stem cell markers, such as CD73, CD90, and CD105, but they have no significant difference between CD146^+^ and CD146^–^ cells. This result has been presented in our previous publication [30]. Our data showed that CD146^+^ cells have a longer doubling time than CD146^–^ cells, which agrees with Espagnolle et al. [23, 41]. Their data show TGFβ1 treatment decreased MSC proliferation and enhanced CD146 expression. The endogenous TGFβ1 might also affect the MSC proliferation. Our data showed the CD146^+^ cells expressed higher TGFβ1 than CD146^–^ cells, Moreover, the CD146^+^ MSCs share some properties with vascular smooth muscle cell, and so the CD146^+^ cells might have better potential for contraction of collagen matrix than CD146^–^ cells. In our data, the CD146^+^ cells have longer doubling time than CD146^–^ cells, but both cells have similar differentiation on chondrogenesis and osteogenesis.

It has been known that the proinflammatory cytokine TNFα can suppress chondrogenesis and osteogenesis in MSCs [43, 44]. In our study, the potential for chondrogenesis and osteogenesis did not differ between CD146^+^ and CD146^–^ cells under normal conditions. By contrast, we also found that inflammation can suppress the effects of both CD146^+^ and CD146^–^ cells on osteogenesis and that the chondrogenic and osteogenic potential of both subpopulations was reduced in cells exposed to TNFα or RASF-conditioned medium. We plan future experiments using qPCR and western blotting to assess the expression of osteo-related genes to confirm the role of TNFα in determining the effects of CD146^+^ cells on cartilage.

The proinflammatory cytokines IL-1β and TNFα can induce MSCs to produce IL-6, which might promote the population of IL-17A-secreting cells and secretion of IL-17A [45, 46]. By contrast, MSCs can release immunomodulatory factors such as IL-10 and TGF-β1, which suppress inflammation by inducing Treg cells and suppressing the proliferation of Th17 cells [14, 15]. However, the effects of MSCs on Th17 cells have not been linked to the CD146 antigen. We found that CD146^+^ and CD146^–^ cells expressed similar levels of TGF-β1, but that CD146^–^ cells induced the proliferation of Th17 cells. In the inflammatory state, IL-6 is an important proinflammatory cytokine that can promote the differentiation of naive T cells into Th17 cells, suppress the differentiation of Treg cells, and accelerate the progression of experimental arthritis [47]. Our data suggest that these differences in activities between CD146^+^ and CD146^–^ MSCs may be related to IL-6 production. An increase in IL-6 production may suppress the proliferation of Treg cells and promote the maturation of Th17 cells.

We found that treatment with TNFα significantly increased IL-6 expression in CD146^–^ MSCs. Coculture of TNFα-treated CD146^–^ cells also showed that CD146^–^ cells significantly activated Th17 cells and increased the production of IL-17. These findings were confirmed by our experiments in the mouse CIA model. TNFα activated the Th17 cell population by promoting IL-6 secretion by CD146^–^ cells, but CD146^+^ cells did not activate the Th17 population. However, the effect of CD146 antigen on MSCs remains unclear. In future research, we will use quantitative PCR to investigate the role of CD146 in stem cells, specifically whether CD146 affects the inflammatory response by modulating genes for inflammatory mediators such as NF-κB and AP-1.

The number of CD4^+^ T cells decreased significantly during coculture with CD146^+^ cells but not during coculture with CD146^–^ cells (Fig. 4a). Our data suggest that both subpopulations of stem cells may have different properties related to the mediation of T-cell function. Recent studies have reported a high concentration of CD146 in RASF [48, 49]. CD146 expression on the cell surface might be involved in interactions between T cells and endothelial cells [50, 51]. We found that neutralization of CD146 by anti-CD146 antibody increased the population of Th17 cells. These results suggest that the interaction of CD146^+^ MSCs with Th17 cells may suppress Th17-mediated cellular processes through the binding of CD146 or other cell adhesion molecules. The local protective effects of CD146 might be similar to those reported for the IL-1 receptor antagonist in RA patients [52].

Kehoe et al. and Swart et al. have shown that stem cells are retained in the joints after IA injection over several weeks [12, 53]. These cells can reduce disease activity or protect the cartilage from damage by directly homing to the sites of lesions. This protection might be due to MSC decreased the Th17 activity in the synovium, and then suppressed the systemic effects of IL-17. In our study, the stem cells were detectable 2 weeks after injection. The increase in IL-17A concentration was found only in the synovium, which suggests that the immune modulation may have local effects after IA injection of stem cells. In terms of cartilage repair, immunofluorescent staining showed that the injected CD146^+^ MSCs homed to cartilage. The mice treated with CD146^+^ cells showed significant improvements in disease progression, and the attachment of CD146^+^ cells to the cartilage preserved proteoglycans, which suggests a possible role of these stem cells in cartilage repair. The cartilage degradation scores did not differ significantly between the saline control and CD146^+^ cell-injected groups. Some reports have shown that synovial inflammation can also affect cartilage repair induced by stem cell therapy [54, 55]. Thus, combined treatment with CD146^+^ cells and anti-tumor necrosis factor drugs might be helpful for cartilage repair. To provide clinical data, grip strength or the indentation test on cartilage may be needed to confirm that the transfused stem cells are active in chondrogenesis [56, 57].

## Conclusions

The expression of CD146 by MSCs seems to play an important role in determining their stem cell properties. CD146^+^ and CD146^–^ stem cells differed in terms of their cell differentiation and immunomodulatory effects. Our data suggest that CD146^+^ MSCs are more capable of promoting immunosuppression than are CD146^–^ cells and that part of this effect seems to involve the suppression of Th17 cells. The lack of CD146 antigen might weaken Th17 suppression in MSCs. Our in vitro and in vivo data also suggest that CD146^+^ MSCs may have greater therapeutic potential than CD146^–^ cells in treating inflammatory arthritis.

## Abbreviations

BrdU: 5-Bromo-2′-deoxyuridine
CD146: Melanoma cell adhesion molecule
CIA: Collagen-induced arthritis
CO_2_: Carbon dioxide
DMEM: Dulbecco’s modified Eagle’s medium
ELISA: Enzyme-linked immunosorbent assay
FBS: Fetal bovine serum
FITC: Fluorescein isothiocyanate
HLA: Human leukocyte antigen
HUMSC: Human umbilical cord-derived mesenchymal stem cell
IA: Intra-articular
IL: Interleukin
MSC: Mesenchymal stem cell
PBS: Phosphate-buffered saline
PE: Phycoerythrin
RA: Rheumatoid arthritis
RASF: Synovial fluid from rheumatoid arthritis patients
SEM: Standard error of the mean
TGF-β: Transforming growth factor beta
Th17: T-helper type 17
TNFα: Tumor necrosis factor alpha
Treg: Regulatory T

## References

[1] Goldring SR (2003). Pathogenesis of bone and cartilage destruction in rheumatoid arthritis. Rheumatology..

[2] Leong DJ, Hardin JA, Cobelli NJ, Sun HB. Mechanotransduction and cartilage integrity. Ann N Y Acad Sci. 2011;1240:32–7. http://www.ncbi.nlm.nih.gov/pubmed/22172037. 10.1111/j.1749-6632.2011.06301.x PMC5007871 22172037

[3] Tetta C, Camussi G, Modena V, Di Vittorio C, Baglioni C (1990). Tumour necrosis factor in serum and synovial fluid of patients with active and severe rheumatoid arthritis. Ann Rheum Dis.

[4] Steiner G, Tohidast-Akrad M, Witzmann G, Vesely M, Studnicka-Benke A, Gal A (1999). Cytokine production by synovial T cells in rheumatoid arthritis. Rheumatology (Oxford).

[5] Eberhard BA, Laxer RM, Andersson U, Silverman ED (1994). Local synthesis of both macrophage and T cell cytokines by synovial fluid cells from children with juvenile rheumatoid arthritis. Clin Exp Immunol.

[6] Moelants EA, Mortier A, Van Damme J, Proost P (2013). Regulation of TNF-alpha with a focus on rheumatoid arthritis. Immunol Cell Biol.

[7] van den Berg WB, Miossec P (2009). IL-17 as a future therapeutic target for rheumatoid arthritis. Nat Rev Rheumatol.

[8] van Hamburg JP, Asmawidjaja PS, Davelaar N, Mus AM, Colin EM, Hazes JM (2011). Th17 cells, but not Th1 cells, from patients with early rheumatoid arthritis are potent inducers of matrix metalloproteinases and proinflammatory cytokines upon synovial fibroblast interaction, including autocrine interleukin-17A production. Arthritis Rheum.

[9] Leipe J, Grunke M, Dechant C, Reindl C, Kerzendorf U, Schulze-Koops H (2010). Role of Th17 cells in human autoimmune arthritis. Arthritis Rheum.

[10] Arroyo-Villa I, Bautista-Caro MB, Balsa A, Aguado-Acin P, Nuno L, Bonilla-Hernan MG (2012). Frequency of Th17 CD4+ T cells in early rheumatoid arthritis: a marker of anti-CCP seropositivity. PloS One.

[11] Augello A, Tasso R, Negrini SM, Cancedda R, Pennesi G (2007). Cell therapy using allogeneic bone marrow mesenchymal stem cells prevents tissue damage in collagen-induced arthritis. Arthritis Rheum.

[12] Kehoe O, Cartwright A, Askari A, El Haj AJ, Middleton J (2014). Intra-articular injection of mesenchymal stem cells leads to reduced inflammation and cartilage damage in murine antigen-induced arthritis. J Transl Med..

[13] Dominici M, Le Blanc K, Mueller I, Slaper-Cortenbach I, Marini F, Krause D (2006). Minimal criteria for defining multipotent mesenchymal stromal cells. The International Society for Cellular Therapy position statement. Cytotherapy.

[14] Pittenger MF, Mackay AM, Beck SC, Jaiswal RK, Douglas R, Mosca JD (1999). Multilineage potential of adult human mesenchymal stem cells. Science.

[15] Aggarwal S, Pittenger MF (2005). Human mesenchymal stem cells modulate allogeneic immune cell responses. Blood.

[16] Gonzalez MA, Gonzalez-Rey E, Rico L, Buscher D, Delgado M (2009). Treatment of experimental arthritis by inducing immune tolerance with human adipose-derived mesenchymal stem cells. Arthritis Rheum.

[17] Despoix N, Walzer T, Jouve N, Blot-Chabaud M, Bardin N, Paul P (2008). Mouse CD146/MCAM is a marker of natural killer cell maturation. Eur J Immunol.

[18] Elshal MF, Khan SS, Raghavachari N, Takahashi Y, Barb J, Bailey JJ (2007). A unique population of effector memory lymphocytes identified by CD146 having a distinct immunophenotypic and genomic profile. BMC Immunol..

[19] Pickl WF, Majdic O, Fischer GF, Petzelbauer P, Fae I, Waclavicek M (1997). MUC18/MCAM (CD146), an activation antigen of human T lymphocytes. J Immunol.

[20] Bardin N, Anfosso F, Masse JM, Cramer E, Sabatier F, Le Bivic A (2001). Identification of CD146 as a component of the endothelial junction involved in the control of cell-cell cohesion. Blood.

[21] Luo Y, Zheng C, Zhang J, Lu D, Zhuang J, Xing S (2012). Recognition of CD146 as an ERM-binding protein offers novel mechanisms for melanoma cell migration. Oncogene.

[22] Shi S, Gronthos S (2003). Perivascular niche of postnatal mesenchymal stem cells in human bone marrow and dental pulp. J Bone Miner Res.

[23] Sacchetti B, Funari A, Michienzi S, Di Cesare S, Piersanti S, Saggio I (2007). Self-renewing osteoprogenitors in bone marrow sinusoids can organize a hematopoietic microenvironment. Cell.

[24] Dmitrieva RI, Minullina IR, Bilibina AA, Tarasova OV, Anisimov SV, Zaritskey AY (2012). Bone marrow- and subcutaneous adipose tissue-derived mesenchymal stem cells: differences and similarities. Cell Cycle.

[25] Martin-Rendon E, Sweeney D, Lu F, Girdlestone J, Navarrete C, Watt SM (2008). 5-Azacytidine-treated human mesenchymal stem/progenitor cells derived from umbilical cord, cord blood and bone marrow do not generate cardiomyocytes in vitro at high frequencies. Vox Sang.

[26] Crisan M, Yap S, Casteilla L, Chen CW, Corselli M, Park TS (2008). A perivascular origin for mesenchymal stem cells in multiple human organs. Cell Stem Cell.

[27] Blocki A, Wang YT, Koch M, Peh P, Beyer S, Law P (2013). Not all MSCs can act as pericytes: functional in vitro assays to distinguish pericytes from other mesenchymal stem cells in angiogenesis. Stem Cells Dev.

[28] Tsang WP, Shu YL, Kwok PL, Zhang FJ, Lee KKH, Tang MK (2013). CD146(+) human umbilical cord perivascular cells maintain stemness under hypoxia and as a cell source for skeletal regeneration. Plos One.

[29] Kouroupis D, Churchman SM, English A, Emery P, Giannoudis PV, McGonagle D (2013). Assessment of umbilical cord tissue as a source of mesenchymal stem cell/endothelial cell mixtures for bone regeneration. Regen Med.

[30] Wu CC, Wu TC, Liu FL, Sytwu HK, Chang DM (2012). TNF-alpha inhibitor reverse the effects of human umbilical cord-derived stem cells on experimental arthritis by increasing immunosuppression. Cell Immunol.

[31] Diekman BO, Wu CL, Louer CR, Furman BD, Huebner JL, Kraus VB (2013). Intra-articular delivery of purified mesenchymal stem cells from C57BL/6 or MRL/MpJ superhealer mice prevents posttraumatic arthritis. Cell Transplant.

[32] Arnett FC, Edworthy SM, Bloch DA, McShane DJ, Fries JF, Cooper NS (1988). The American Rheumatism Association 1987 revised criteria for the classification of rheumatoid arthritis. Arthritis Rheum.

[33] Kretlow JD, Jin YQ, Liu W, Zhang WJ, Hong TH, Zhou GD (2008). Donor age and cell passage affects differentiation potential of murine bone marrow-derived stem cells. BMC Cell Biol..

[34] Yen BL, Chang CJ, Liu KJ, Chen YC, Hu HI, Bai CH (2009). Brief report—human embryonic stem cell-derived mesenchymal progenitors possess strong immunosuppressive effects toward natural killer cells as well as T lymphocytes. Stem Cells.

[35] Gronthos S, Mankani M, Brahim J, Robey PG, Shi S (2000). Postnatal human dental pulp stem cells (DPSCs) in vitro and in vivo. Proc Natl Acad Sci U S A.

[36] Kucerova L, Altanerova V, Matuskova M, Tyciakova S, Altaner C (2007). Adipose tissue-derived human mesenchymal stem cells mediated prodrug cancer gene therapy. Cancer Res.

[37] Karahuseyinoglu S, Cinar O, Kilic E, Kara F, Akay GG, Demiralp DO (2007). Biology of stem cells in human umbilical cord stroma: in situ and in vitro surveys. Stem Cells.

[38] Baksh D, Yao R, Tuan RS (2007). Comparison of proliferative and multilineage differentiation potential of human mesenchymal stem cells derived from umbilical cord and bone marrow. Stem Cells.

[39] Puissant B, Barreau C, Bourin P, Clavel C, Corre J, Bousquet C, et al. Immunomodulatory effect of human adipose tissue-derived adult stem cells: comparison with bone marrow mesenchymal stem cells. Br J Haematol. 2005;129(1):118–29. 10.1111/j.1365-2141.2005.05409.x 15801964

[40] Battula VL, Treml S, Bareiss PM, Gieseke F, Roelofs H, de Zwart P (2009). Isolation of functionally distinct mesenchymal stem cell subsets using antibodies against CD56, CD271, and mesenchymal stem cell antigen-1. Haematologica.

[41] Espagnolle N, Guilloton F, Deschaseaux F, Gadelorge M, Sensebe L, Bourin P (2014). CD146 expression on mesenchymal stem cells is associated with their vascular smooth muscle commitment. J Cell Mol Med.

[42] Tormin A, Li O, Brune JC, Walsh S, Schutz B, Ehinger M (2011). CD146 expression on primary nonhematopoietic bone marrow stem cells is correlated with in situ localization. Blood.

[43] Wehling N, Palmer GD, Pilapil C, Liu F, Wells JW, Muller PE (2009). Interleukin-1beta and tumor necrosis factor alpha inhibit chondrogenesis by human mesenchymal stem cells through NF-kappaB-dependent pathways. Arthritis Rheum.

[44] Zhao L, Huang J, Zhang HW, Wang Y, Matesic LE, Takahata M (2011). Tumor necrosis factor inhibits mesenchymal stem cell differentiation into osteoblasts via the ubiquitin E3 ligase Wwp1. Stem Cells.

[45] Svobodova E, Krulova M, Zajicova A, Pokorna K, Prochazkova J, Trosan P (2012). The role of mouse mesenchymal stem cells in differentiation of naive T-cells into anti-inflammatory regulatory T-cell or proinflammatory helper T-cell 17 population. Stem Cells Dev.

[46] Eljaafari A, Tartelin ML, Aissaoui H, Chevrel G, Osta B, Lavocat F (2012). Bone marrow-derived and synovium-derived mesenchymal cells promote Th17 cell expansion and activation through caspase 1 activation: contribution to the chronicity of rheumatoid arthritis. Arthritis Rheum.

[47] Kimura A, Kishimoto T (2010). IL-6: regulator of Treg/Th17 balance. Eur J Immunol.

[48] Neidhart M, Wehrli R, Bruhlmann P, Michel BA, Gay RE, Gay S (1999). Synovial fluid CD146 (MUC18), a marker for synovial membrane angiogenesis in rheumatoid arthritis. Arthritis Rheum.

[49] Wu C, Goodall JC, Busch R, Gaston JS (2015). Relationship of CD146 expression to secretion of interleukin (IL)-17, IL-22 and interferon-gamma by CD4(+) T cells in patients with inflammatory arthritis. Clin Exp Immunol.

[50] Elshal MF, Khan SS, Takahashi Y, Solomon MA, McCoy JP (2005). CD146 (Mel-CAM), an adhesion marker of endothelial cells, is a novel marker of lymphocyte subset activation in normal peripheral blood. Blood.

[51] Guezguez B, Vigneron P, Lamerant N, Kieda C, Jaffredo T, Dunon D (2007). Dual role of melanoma cell adhesion molecule (MCAM)/CD146 in lymphocyte endothelium interaction: MCAM/CD146 promotes rolling via microvilli induction in lymphocyte and is an endothelial adhesion receptor. J Immunol.

[52] Kay J, Calabrese L (2004). The role of interleukin-1 in the pathogenesis of rheumatoid arthritis. Rheumatology (Oxford).

[53] Swart JF, de Roock S, Hofhuis FM, Rozemuller H, van den Broek T, Moerer P (2015). Mesenchymal stem cell therapy in proteoglycan induced arthritis. Ann Rheum Dis.

[54] Jones E, Churchman SM, English A, Buch MH, Horner EA, Burgoyne CH (2010). Mesenchymal stem cells in rheumatoid synovium: enumeration and functional assessment in relation to synovial inflammation level. Ann Rheum Dis.

[55] Papadopoulou A, Yiangou M, Athanasiou E, Zogas N, Kaloyannidis P, Batsis I (2012). Mesenchymal stem cells are conditionally therapeutic in preclinical models of rheumatoid arthritis. Ann Rheum Dis.

[56] Allen KD, Griffin TM, Rodriguiz RM, Wetsel WC, Kraus VB, Huebner JL (2009). Decreased physical function and increased pain sensitivity in mice deficient for type IX collagen. Arthritis Rheum.

[57] Bae WC, Temple MM, Amiel D, Coutts RD, Niederauer GG, Sah RL (2003). Indentation testing of human cartilage: sensitivity to articular surface degeneration. Arthritis Rheum.

